# In Vitro Anti-diabetic Activity of Pomegranate Peel Extract-Mediated Strontium Nanoparticles

**DOI:** 10.7759/cureus.51356

**Published:** 2023-12-30

**Authors:** Parameswari Royapuram Parthasarathy, Ilammaran Varshan E, Rajeshkumar Shanmugam

**Affiliations:** 1 Pharmacology, Centre for Transdisciplinary Research, Saveetha Dental College and Hospitals, Saveetha Institute of Medical and Technical Sciences, Saveetha University, Chennai, IND; 2 Pharmacology, Centre for Global Health Research, Saveetha Medical College and Hospitals, Saveetha Institute of Medical and Technical Sciences, Saveetha University, Chennai, IND; 3 Dentistry, Saveetha Dental College and Hospitals, Saveetha Institute of Medical and Technical Sciences, Saveetha University, Chennai, IND

**Keywords:** type 2 diabetes mellitus, strontium, pomegranate peel, nanoparticles, α-glucosidase, α-amylase

## Abstract

Introduction

Type 2 diabetes mellitus and its associated health complications represent a significant public health issue due to its wide prevalence. The primary disadvantages of current oral anti-diabetic drugs are their limited bio-availability and their quick release, which necessitates more frequent dosing. Similar limitations are encountered when using natural products for the management of type 2 diabetes. Consequently, nanoparticles have been developed with the goal of enhancing the physicochemical stability of the drugs, thereby improving their bio-availability. In view of the given background, the present study aimed to explore the efficacy of strontium nanoparticles derived from pomegranate peel extract in managing type 2 diabetes mellitus.

Materials and methods

The aqueous extract of pomegranate peel was prepared using standard protocol and the strontium nanoparticles were prepared by green synthesis method using pomegranate peel extract. The prepared aqueous extract of pomegranate peel and the nanoparticles were assessed for their inhibitory potential against α-amylase and α-glucosidase enzymes activity by 3,5-dinitrosalicylic acid (DNSA) and p-nitro-phenyl-ɑ-D glucopyranoside (p-NPG) assays, respectively.

Results

The pomegranate peel-mediated strontium nanoparticles (PP-Sr NPs) and standard acarbose were assessed for their inhibitory effect against diabetic enzymes, α-amylase, and α-glucosidase at different concentrations range of 5-160 μg/ml. The results showed that PP-Sr NPs exhibited a maximum inhibition of 79.28% and 76.17% against α-amylase and α-glucosidase respectively at the highest concentration of 160 μg/ml. Acarbose showed a maximum inhibition of 88.02% and 84.47% against α-amylase and α-glucosidase respectively at 160 μg/ml. The inhibitory effect of the PP-Sr NPs and standard acarbose was found to be concentration-dependent.

Conclusion

From the results, it is concluded that the PP-Sr NPs may be useful for decreasing postprandial glucose levels. Further studies using in vitro cell lines and in vivo diabetic models may substantiate the antidiabetic potential of PP-Sr NPs.

## Introduction

Diabetes mellitus (DM) is a metabolic disorder characterized by chronic high blood sugar levels that occur due to insulin dysfunction and impaired secretion, which are responsible for processing carbohydrates, proteins, and fats. The International Diabetes Federation estimates that there are currently 40.9 million people with diabetes worldwide, and this number is expected to increase to 60.9 million by 2025 and possibly quadruple by 2030 [1]. There are two main forms of diabetes, known as type 1 diabetes mellitus (T1DM) and type 2 diabetes mellitus (T2DM). T1DM is characterized by an autoimmune process that damages the pancreatic cells responsible for producing insulin, leading to a decrease in insulin production. On the other hand, T2DM results from dysfunction in pancreatic beta cells, which affects the body's ability to effectively utilize insulin [2]. T2DM represents the majority form of diabetes with approximately 90% of total diabetes cases and has emerged as a significant global health concern [3]. Research has shown that elevated blood sugar levels after meals, known as postprandial hyperglycemia (PPHG), play a significant role in the development of T2DM and its associated complications [4]. Enzymes like α-amylase and α-glucosidase are responsible for breaking down carbohydrates into smaller molecules, and inhibiting these enzymes is considered an important strategy for managing PPHG [5]. Therefore, developing inhibitors that target these enzymes can help prevent spikes in blood glucose levels and effectively control PPHG.

Although carbohydrate hydrolyzing enzyme inhibitors such as acarbose, miglitol, and voglibose are available for managing T2DM, their long-term use is associated with significant side effects such as gastrointestinal discomfort, severe hypoglycemia, and undesired weight gain [6]. Additionally, the prolonged use of chemical or synthetic inhibitors presents certain constraints such as suboptimal or ineffective dosing strategies, reduced drug efficacy, a lack of precise target specificity, and challenges related to drug solubility and permeability [7]. Consequently, the search for alternative enzyme inhibitors with negligible side effects continues to be a persistent effort in the field of T2DM management [8]. In this context, nanotechnology-based strategies hold substantial promise in terms of enhancing therapeutic effectiveness and augmenting the quality of life for patients. Earlier studies have shown that nanoparticles possess unique properties that aid in overcoming the challenges posed by modern anti-diabetic medicines [9,10].

Over the past decade, nanoparticles have garnered significant attention due to their distinct optical, electronic, and physicochemical characteristics. One of the methods for synthesizing nanoparticles that has gained prominence is the green synthesis approach, known for its non-hazardous, eco-friendly, and cost-effective nature. This method involves utilizing plants, fruits, vegetables, and microorganisms to create nanoparticles [11]. However, as the demand for nanoparticles continues to rise, there is a need to explore alternative resources for their synthesis that do not harm the environment, are less time-consuming, and are economically viable. In the realm of nanoparticle synthesis, the eco-friendly approach of using fruit pulp and peel extracts as well as other plant components as a reducing medium is preferred over traditional chemical methods [12]. Therefore, biowastes such as seeds, fruit/vegetable peels, and other agricultural waste materials that are enriched with bioactive compounds like polyphenols, flavonoids, polysaccharides, lignans, etc., have been employed as raw materials for nanoparticle synthesis [13, 14].

Pomegranates (Punica granatum L.), belonging to the Lythraceae family, are known for their various health benefits, including the medicinal properties of the fruit's rind [15]. Pomegranate peel and other parts of the fruit contain approximately 48 phenolic compounds, including flavonoids, anthocyanidins, condensed tannins, and hydrolyzable tannins [16, 17]. Studies indicate that polyphenols, including hydrolyzed tannins like punicalagin and punicalin, may be responsible for the fruit's antioxidant, anti-diabetic, anti-obesity, anti-inflammatory, and other beneficial effects [16]. Moreover, the peel of pomegranate has also been reported for its significant anti-diabetic effect in terms of improving insulin sensitivity, reduction of glucose levels, and amelioration of oxidative stress in diabetic mouse models [16-20]. Nevertheless, there is a paucity of research regarding both the efficacy of utilizing pomegranate peel extract in nanoparticle synthesis and its potential pharmacological impacts. Therefore the aim of the present study is to evaluate the potential of pomegranate peel extract for synthesis of strontium nanoparticles and its in vitro inhibitory effect on enzymes involved in carbohydrate digestion.

## Materials and methods

Study design 

The study employed a laboratory-based in vitro experimental design involving the preparation of aqueous extract from pomegranate peel using a standardized protocol. Strontium nanoparticles were synthesized via a green synthesis method facilitated by the pomegranate peel extract. The strontium nanoparticles were evaluated for their in vitro enzyme inhibition potential against diabetic enzymes. The inhibitory activity against α-amylase and α-glucosidase enzymes was assessed using 3,5-dinitrosalicylic acid and p-nitro-phenyl-ɑ-D glucopyranoside assays, respectively. Acarbose was used as the standard for both assays. The synthesized strontium nanoparticles and the standard drug acarbose were serially diluted to six different concentrations of 5, 10, 20, 40, 80, and 160 μg/ml using distilled water. Each concentration was tested in triplicate (n=3/concentration) for accurate statistical analysis. Subsequently, the prepared pomegranate peel extract-mediated strontium nanoparticles (PP-Sr NPs) and acarbose were evaluated to observe their inhibitory effects across various concentration ranges.

Preparation of pomegranate peel extract

The process involved taking the skin of the Punica granatum L. fruit (commonly known as pomegranate), drying it in the shade, and then grinding it into a coarse powder. A portion of this coarse powder, weighing 1.025 g, was measured and dissolved in 100 ml of distilled water, which was then heated to 90°C and allowed to boil for 15 minutes. Later, the boiled pomegranate peel was filtered through Whatman No. 1 filter paper.

Preparation of strontium nanoparticles

Strontium nitrate (1.0 g) was measured and mixed with 50 ml of distilled water in a conical flask. Subsequently, 50 ml of boiled pomegranate peel extract was added to the strontium nitrate mixture using a magnetic stirrer. This mixture was then placed in an orbital shaker for a duration of 24 hours. After the incubation period, the contents were transferred into a 15 ml falcon tube and centrifuged at 8000 rotations per minute (rpm) for 15 minutes. Following centrifugation, the resulting pellets were extracted from the centrifuge tube and subjected to heating at 100°C in a hot air oven for a full day. The resulting dried PP-Sr NPs were then examined for their potential against inhibition of carbohydrate hydrolyzing enzymes within in vitro conditions. 

α-amylase inhibitory activity

The study on inhibiting α-amylase in vitro followed Wickramaratne et al. 2016 method [21] wherein 100μL of different concentrations (5, 10, 20, 40, 80, and 160 μg/ml) of PP-Sr NPs were taken in different test tubes. Then, 200 μL of α-amylase enzyme (HiMedia RM638) and 100 μL of 2mM phosphate buffer (pH-6.9) were added to all the tubes. After a 20-minute incubation, 100 μL of 1% starch solution was added. The same procedure was carried out for control samples with 200 μL phosphate buffer without the addition of enzyme. After a five-minute incubation, 500 μL of dinitrosalicylic acid reagent was added to both the control and test samples. They were then placed in a boiling water bath for five minutes. The absorbance was measured at 540 nm using a spectrophotometer and the percentage of α-amylase enzyme inhibition was calculated using this formula:

% inhibition = [(Control-Test)/Control] *100

α-glucosidase inhibitory activity

The inhibition of the enzyme α-glucosidase was assessed following a method originally outlined by Sancheti et al. in 2011, with minor modifications [22]. In this procedure, a reaction mixture was prepared by combining 50 μL of 0.1 M phosphate buffer (pH 7.0), 25 μL of 0.5 mM 4-nitrophenyl α-D-glucopyranoside (dissolved in 0.1 M phosphate buffer, pH 7.0), 10 μL of varying concentrations (5, 10, 20, 40, 80, and 160 μg/ml) of PP-Sr NPs, and 25 μL of α-glucosidase solution. The α-glucosidase solution was prepared by diluting a stock solution of 1 mg/mL in 0.01 M phosphate buffer (pH 7.0) to a final concentration of 0.1 Unit/mL with the same buffer, just before the assay. This reaction mixture was then incubated at 37°C for 30 minutes. To stop the reaction, 100 μL of 0.2 M sodium carbonate solution was added. The enzymatic breakdown of the substrate was assessed by measuring the amount of p-nitrophenol released in the reaction mixture at 410 nm using a microplate reader. Blank samples were also prepared to correct for background absorbance, where enzymes were substituted with buffers. Control experiments were carried out in the same manner, replacing the plant extracts with methanol. Acarbose served as a positive control in these experiments. All tests were conducted in triplicate.

Statistical analysis

The data obtained for α-amylase and α-glucosidase inhibition assays were analyzed by Student's t-test using SPSS software (IBM Corp., Armonk, NY). For statistical analysis, the triplicate values of each single concentration were taken into account and a comparison was done between nanoparticle and standard. The % inhibitory effect (n=3/concentration) was represented as mean±SEM for triplicates. P<0.05 is considered as significant. 

## Results

**Figure 1:**
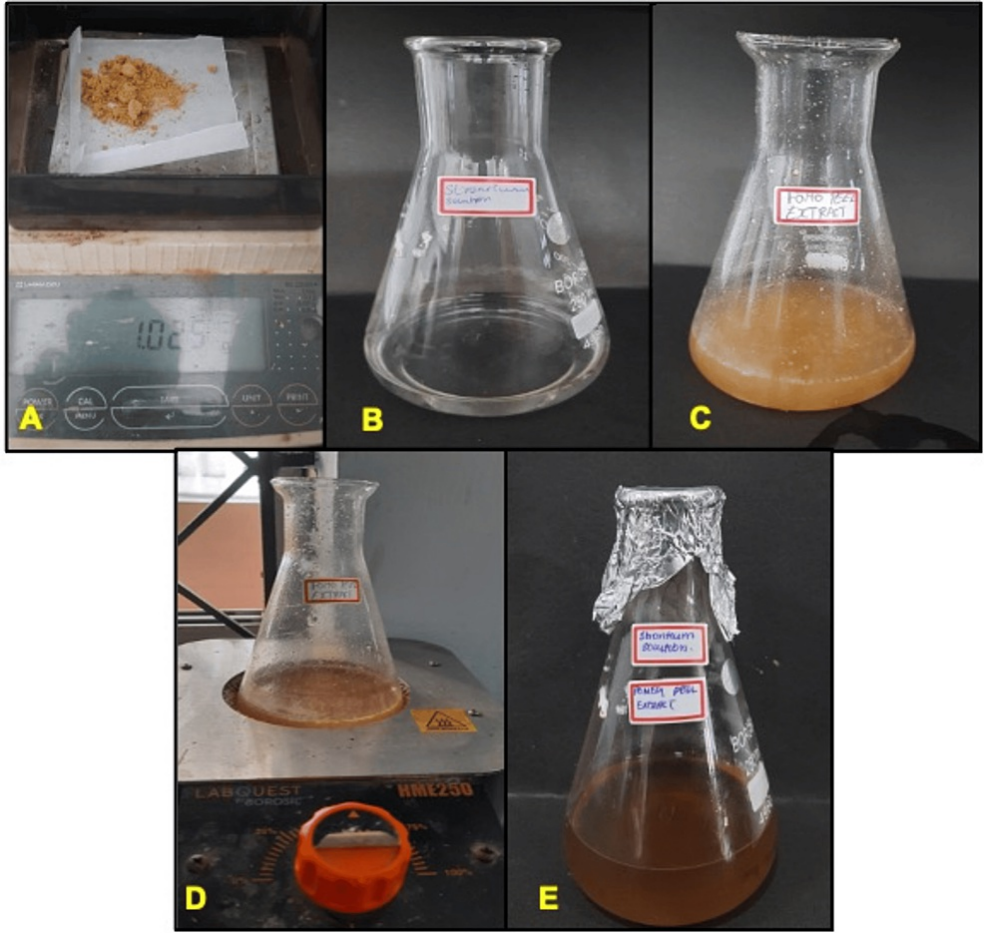
Preparation steps involved in pomegranate peel extract-mediated synthesis of strontium nanoparticles. A: strontium nitrate; B: 1.0g of strontium nitrate mixed with 50 ml of distilled water; C: pomegranate peel extract; D: boiling of pomegranate peel extract; E: pomegranate peel extract mediated strontium nanoparticle solution.

The present study evaluated the antidiabetic effect of PP-Sr NPs by in vitro methods. The synthesized strontium nanoparticles were observed to note any color change. In the present study, pomegranate peel extract mixed with strontium solution showed a color change from pale yellow to dark brown, indicating strontium ion reduction and formation of nanoparticles (Figure 1). The formation of strontium nanoparticles was confirmed by UV-visible peak absorbance at 203nm. After confirming the formation of PP-Sr NPs, the effect of nanoparticles on α-amylase and α-glucosidase enzyme activity was observed.

Effect of PP-Sr NPs on a-amylase activity

**Figure 2:**
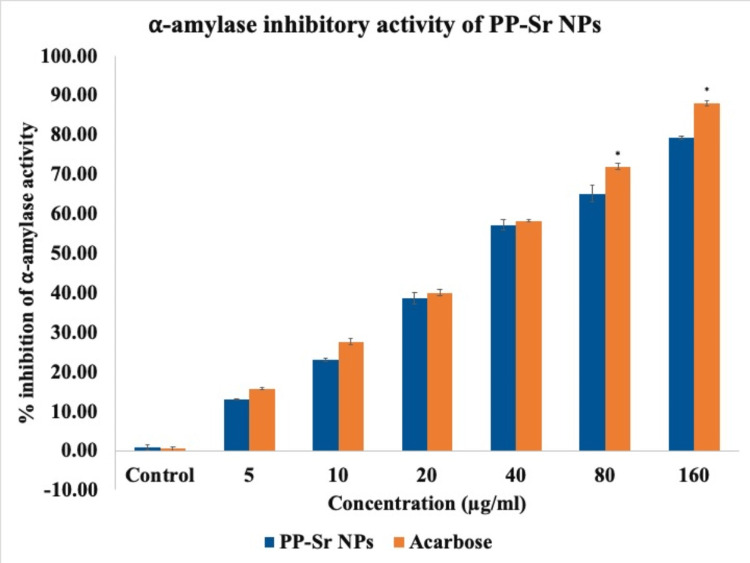
Bar graph representing α-amylase inhibitory activity of PP-Sr NPs against α-amylase enzyme in vitro compared with standard acarbose. The blue bar represents different concentrations of PP-Sr NPs. The orange bar represents different concentrations of acarbose. The X-axis represents the concentration of PP-Sr NPs and the Y-axis represents the % inhibition of α-amylase. PP-Sr NPs: pomegranate peel extract-mediated strontium nanoparticles

The effect of PP-Sr NPs on the activity of α-amylase enzyme was evaluated. The results showed that the strontium nanoparticles effectively inhibited the amylase enzyme activity with a maximum inhibition of 79% at a concentration of 160μg/ml (Figure 2). The IC_50_ (the concentration required for 50% inhibition) was determined to be 38.07μg/ml, which was slightly higher than the standard drug acarbose, which had an IC_50_ of 34.21μg/ml. 

Effect of PP-Sr NPs on α-glucosidase activity

**Figure 3:**
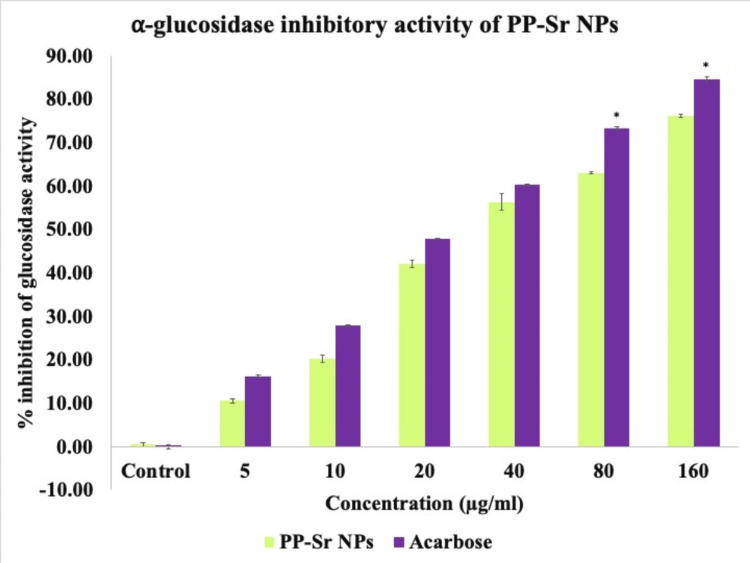
Bar graph representing α-glucosidase inhibitory activity of PP-Sr NPs against α-glucosidase enzyme in vitro compared with standard acarbose. The green bar represents different concentrations of PP-Sr NPs. The purple bar represents different concentrations of acarbose. The X-axis represents the concentration of PP-Sr NPs and the Y-axis represents the % inhibition of α-glucosidase. PP-Sr NPs: pomegranate peel extract-mediated strontium nanoparticles

A significant inhibitory effect was observed for the α-glucosidase enzyme, with a maximum inhibition of 76% at the highest concentration of 160μg/ml (Figure 3). The IC_50_ value for PP-Sr NPs was 30.56μg/ml, while the standard drug acarbose had an IC_50_ of 29.42μg/ml. These findings suggest that the inhibition of these pancreatic enzymes by PP-Sr NPs can delay the absorption of carbohydrates, making them potentially useful in managing postprandial hyperglycemia (PPHG). 

## Discussion

The present study aimed to investigate the anti-diabetic potential of strontium nanoparticles synthesized using pomegranate peel extract. The results of our study showed a significant reduction in the activity of α-amylase and α-glucosidase enzymes by strontium nanoparticles derived from pomegranate peel extract. By inhibiting these digestive enzymes, the intestinal absorption of glucose can be slowed down, which is an effective strategy for managing type 2 diabetes. Delaying the absorption of glucose in the intestine helps control the increase in post-meal glucose levels [5]. Previous research has demonstrated the anti-diabetic potential of various metal and metal oxide nanoparticles, including silver nanoparticles synthesized from Salmonella enterica, which exhibited marked inhibition against carbohydrate metabolizing enzymes, comparable to that of standard acarbose, and was found to be non-toxic [10, 23, 24]. Additionally, silver nanoparticles synthesized from various plant sources, such as the whole plant of Pterocarpus marsupium, leaves of Cassia auriculata, and leaves of Annona muricata, have also shown significant anti-diabetic properties by inhibiting α-amylase and α-glucosidase enzymes [25-27]. Another study demonstrated the anti-diabetic potential of zinc oxide nanoparticles synthesized using citrus lemon root [28]. Rubidium-doped indium sulfide nanoparticles have also shown marked inhibition of α-glucosidase [29]. Furthermore, previous research has highlighted the therapeutic potential of strontium nanoparticles in addressing diabetes-related physiological changes by regulating insulin release in high-fat diet-induced Drosophila models [30]. Our findings support the potential anti-diabetic effects of strontium nanoparticles, suggesting that the biosynthesized strontium nanoparticles may help mitigate T2DM by influencing the carbohydrate metabolism pathway.

Previous research findings have demonstrated the significant potential of pomegranate peel extract in addressing diabetes, both in preclinical and clinical models [16-19]. In a study focusing on diabetic cardiomyopathy, pomegranate peel extract was observed to enhance lipid profiles and cardiac indicators, such as troponin, while concurrently reducing oxidative stress in a rat model [17]. Another study revealed that the administration of pomegranate peel extract effectively prevented glucose intolerance, insulin resistance, and oxidative stress while decreasing levels of amylase and lipase in rats subjected to a high-fructose diet [18]. Similarly, when rats were provided with pomegranate peel extract in conjunction with L-carnitine over a 12-week period, it significantly lowered total cholesterol, triglycerides, and low-density lipoprotein levels. Moreover, this combination therapy regulated oxidative stress by reducing lipid peroxide levels and increasing antioxidants in streptozotocin-induced diabetic rats [19]. A randomized clinical study also reported positive outcomes from the use of capsules containing pomegranate peel extract in type 2 diabetic patients. This study found that an eight-week supplementation with these capsules significantly reduced systolic and diastolic blood pressure in type 2 diabetes patients. Additionally, the treatment had favorable effects on the levels of triglycerides, high-density lipoprotein cholesterol, thiobarbituric acid reactive substances (TBARS), hemoglobin A1c (HbA1c), and fatty acid profiles in the total plasma lipids of these patients, suggesting the potential of pomegranate peel extract to have hypolipemic, hypoglycemic, and antioxidative benefits [16]. Nevertheless, all these studies have employed the whole crude pomegranate peel extract. The present investigation utilized pomegranate peel extract for the synthesis of strontium nanoparticles and has additionally revealed the considerable anti-diabetic effects of these strontium nanoparticles within in vitro conditions. 

The beneficial impact of pomegranate peel extract on diabetes management might be attributed to its tannin content [14]. More specifically, pomegranate peel contains hydrolyzable tannins known as punicalagin. These punicalagins undergo hydrolysis during the digestive process, transforming into ellagic acid, which is further metabolized into urolithins by intestinal microorganisms [15]. These naturally occurring compounds play a role in regulating glucose release in a gradual and steady manner, potentially leading to better control of blood glucose levels and a reduced glycemic index [5]. The present study results are consistent with previous research, affirming the promising potential of strontium nanoparticles derived from pomegranate peel in the context of diabetes management.

Limitations

The preclinical assessment conducted is a preliminary evaluation, and additional research is necessary to uncover the molecular mechanism responsible for the nanoparticles' anti-diabetic properties. Furthermore, in-depth in vivo studies are needed to confirm and strengthen the evidence supporting the synthesized nanoparticles' effectiveness in treating diabetes. 

## Conclusions

Strontium nanoparticles have been successfully synthesized from pomegranate peel extract. The synthesized PP-SR NPs were found to possess significant anti-diabetic properties, namely, a marked inhibition of carbohydrate hydrolyzing enzymes. PP-Sr NPs, which are derived naturally and feature an effective drug delivery system with minimal side effects, have the potential to be further developed as a potent anti-diabetic medication with additional research.
